# Attitudes and Acceptance of COVID-19 Vaccination Among Nurses and Midwives in Cyprus: A Cross-Sectional Survey

**DOI:** 10.3389/fpubh.2021.656138

**Published:** 2021-06-16

**Authors:** Georgia Fakonti, Maria Kyprianidou, Giannos Toumbis, Konstantinos Giannakou

**Affiliations:** ^1^Department of Health Sciences, School of Sciences, European University Cyprus, Nicosia, Cyprus; ^2^Cyprus International Institute for Environmental and Public Health, Cyprus University of Technology, Limassol, Cyprus

**Keywords:** COVID-19 vaccines, COVID-19, mass vaccination, vaccination, nurses, vaccines, vaccination refusal

## Abstract

Healthcare workers are at the frontline of the COVID-19 pandemic and have been identified as a priority target group for COVID-19 vaccines. This study aimed to determine the COVID-19 vaccination intention among nurses and midwives in Cyprus and reveal the influential factors that affected their decision. An Internet-based cross-sectional survey was conducted between December 8 and 28, 2020. Data collection was accomplished using a self-administered questionnaire with questions about socio-demographic characteristics, questions assessing general vaccination-related intentions and behaviors, and the intention to accept COVID-19 vaccination. A sample of 437 responders answered the survey, with 93% being nurses and 7% midwives. A small proportion of the participants would accept a vaccine against COVID-19, while 70% could be qualified as “vaccine hesitant.” The main reasons for not receiving the COVID-19 vaccine were concerns about the vaccine's expedited development and fear of side effects. More females, individuals with a larger median age, and a higher number of years of working experience, intended to accept the COVID-19 vaccination, compared with those not intended to accept and undecided groups (*p* < 0.01). Having a seasonal flu vaccination in the last 5 years, receiving the vaccines recommended for health professionals, and working in the private sector were associated with a higher probability of COVID-19 vaccination acceptance. A considerable rate of nurses and midwives in Cyprus reported unwillingness to receive a COVID-19 vaccine due to vaccine-related concerns. Our findings highlight the need for forthcoming vaccination campaigns and programs to tackle coronavirus vaccine hesitancy barriers to achieve the desirable vaccination coverage.

## Introduction

At the end of 2019, cases of pneumonia with unknown etiology were reported in Wuhan, China (1). Scientists identified the pathological agent responsible for the disease as a novel coronavirus (SARS-CoV-2), and they named the causing disease COVID-19 (2). Within a short time, the virus rapidly spread worldwide, and in March 2020, the World Health Organization (WHO) declared COVID-19 as a pandemic (3, 4). Since then, significant efforts to identify new cases (5, 6) and regulations to control viral transmission have been made globally. Researches attempted to discover drugs for treatment (7, 8), while an enormous number of laboratories aimed to develop vaccines for disease prevention.

It was widely recognized in the scientific community that vaccination is a key intervention to cope with the pandemic. Vaccination is one of the most significant public health achievements (9, 10). Vaccines prevent over 20 fatal diseases, and immunization saves approximately 2–3 million lives yearly (11). Historically the timeframe on vaccine development is a long stretch; however, novel technological advances reduced the developmental time (12, 13). The advances, along with available prior research on coronaviruses, the global emergency, and the vast funding for research, contribute to a high-speed COVID-19 vaccine development (14, 15). For COVID-19 vaccine, more than 230 vaccine candidates were introduced in pre-clinical and clinical phases using both classical and next-generation platforms (16).

The excessive effort for COVID-19 vaccine development can be considered successful if the public intention to receive the vaccination is high. Vaccine hesitancy has grown during the last decade and influenced the vaccination coverage (17). Previous research indicated public unwillingness to receive new vaccines during a pandemic (18–20). The factors that determine public decision for vaccination are complex (21–23). WHO proposed the “3C” model of vaccine hesitancy that includes three aspects: confidence, convenience, complacency, and a table with vaccine hesitancy influences; contextual, individual and group; and vaccine/vaccination-related issues (24, 25).

Healthcare workers are still in the front-line of the fight against the new virus, even by risking their own lives (26). A suboptimal vaccination coverage for routine vaccines was identified among healthcare workers across the world (27–30), and thus, their intention to receive the COVID-19 vaccination is important. The literature concerning the healthcare worker's acceptability of COVID-19 vaccination is currently limited; however, most of the examined studies have shown controversial results. Studies in Greece (31) and the Democratic Republic of the Congo (32) revealed a minor proportion of healthcare workers willing to be vaccinated against COVID-19. Nurses' unwillingness to receive COVID-19 vaccination was also reported in surveys in China (33). In contrast, a high percentage of healthcare workers in France (34) and nurses in Hong Kong (35) were willing to be vaccinated. Various factors could influence the COVID-19 vaccine acceptance comprising the infection risk and work in COVID-19 departments (36, 37).

During the last years, Cyprus's national health system has changed dramatically by integrating the public and private health sectors to achieve higher public coverage (38). The integration of health resources could potentially influence vaccination programs; however, the new general healthcare system is still in its infancy. Cyprus's vaccination schedule follows WHO recommendations, and healthcare professionals are encouraged to be vaccinated. The nursing staff represents the largest part of healthcare professionals in Cyprus and often acts as vaccine administrators for the public.

Acceptance toward COVID-19 vaccination is not universal and is influenced by several factors (39). The health protection of healthcare workers such as nurses and midwives is crucial during pandemics; thus, it is essential to estimate the proportion of professionals willing to be vaccinated and identify the specific factors that affected their decision. Therefore, our study aims to investigate the nurses' and midwives' intention to accept COVID-19 vaccination in Cyprus and factors that influence their decision.

## Materials and Methods

### Study Design, Participants, and Procedure

A cross-sectional anonymous online survey was conducted among nurses and midwives in Cyprus between 8 and 28 December 2020 (before introducing COVID-19 vaccination programs in Cyprus). The referent population included registered nurses and midwives working in either public or private service provision. Any inpatient, outpatient, or outreach service in the community setting was eligible for this study. Only nurses and midwives that were in direct contact with patients were eligible to participate; hence, nursing students and nurses working in positions that did not provide direct care were excluded from the study sample. We attempted to keep a similar proportion of registered nurses among the five government-controlled districts of the Republic of Cyprus [Nicosia (46%), Limassol (26%), Larnaca (12%), Paphos (10%), and Ammochostos (6%)], as reported by the Statistical Service of Cyprus (40).

The data were collected using an online questionnaire. The questionnaire was administered using Google Forms and dispersed using social media apps (i.e., Facebook), instant messaging apps (i.e., WhatsApp, Viber, and Messenger), social networking (i.e., LinkedIn), and institutional emails through snowball sampling. An information sheet about the study was included at the beginning of the questionnaire, followed by an electronic consent form. During the survey, no electronic signatures were required, and the IP addresses of participants were not collected. At the end of the survey period, the data were extracted from the survey host and imported into statistical software for analysis. The survey was voluntary, and no incentive was offered. The dissemination of the questionnaire was initiated after the Cyprus National Bioethics Committee (CNBC) approval.

### Instruments and Variables

The online self-administered questionnaire contained 26 closed-ended and multiple-choice questions in Greek language on demographic characteristics (i.e., age, gender, marital and educational status), work-related factors, questions assessing general vaccination-related intentions and behaviors, and questions related to COVID-19 vaccination. The questionnaire was formed on the basis of extensive literature search (33–36, 39, 41). Before the actual study, face validity was tested in a small pilot study of 40 nurses to test the clarity and the applicability of all items of the survey, and to identify any difficulties that may be occurred during data collection. Appropriate changes were made to ensure sample access to representative answers. For example, we realized that it was difficult for participants to give only one reason for not receiving COVID-19 vaccine rather than having the choice of selecting more than one options. The pilot sample was not included in the study sample. The Cronbach's α-value for internal reliability was 0.743 (Supplementary Table 1).

Marital status was classified as unmarried, married/engaged, or separated/divorced/widowed. Educational level was recorded into three categories (those who completed a bachelor's degree, those who completed a master's degree, and those who completed a doctoral degree). Moreover, the number of their children on a scale ranging from 0 to 5 or more was required.

Questions concerning work-related factors covered the service setting they were working in (e.g., intensive care units, accident, emergency department, and others), whether they were working in public or private service provision, and their years of work experience. For the vaccination-related intentions and behaviors, the participants were asked for information regarding whether they accepted seasonal influenza vaccination in the past 5 years (yes, no, or not sure), whether or not they received the vaccines recommended for health professionals, whether or not they know the recommended vaccinations for health professionals, whether or not they were vaccinated at their workplace, if they were not vaccinated at their workplace, where they were vaccinated, if they were vaccinated at their workplace, what were the main reasons for vaccination, whether they consider themselves as being in a vulnerable group (pregnant, diabetic, immunosuppressed, and other) to whom vaccination is recommended, whether or not they attended a conference, seminar, or other training programs on vaccination in the last 2 years, whether they recommended and promoted vaccination in their work, and which were the primary sources of information on vaccination. Furthermore, for the questions relating to the COVID-19 vaccination, participants were asked for information about whether or not they believe nurses and midwives should be vaccinated against COVID-19, whether they intended to accept COVID-19 vaccination when it becomes available, whether or not they intended to accept to vaccinate their children with the COVID-19 vaccine, and what were the main reasons for not receiving the COVID-19 vaccine.

### Ethics Approval

This study was conducted according to the Declaration of Helsinki guidelines, and all procedures involving research study participants were approved by the Cyprus National Bioethics Committee (CNBC) (EEBK EΠ 2020.01.255). The application with the relevant questionnaire outlined the study objectives and outcomes, the data collection process, data management, the data's use, and the expected benefits were submitted to CNBC. Participation was anonymous, and all the participants were informed about the study aim and objectives before participating.

### Statistical Analysis

Continuous variables with normal distribution are presented as mean ± standard deviation (SD), while continuous variables with not normal distribution as median (q_1_, q_3_). The distributions of continuous variables were assessed for normality using the Shapiro–Wilk test. Categorical variables (i.e., sex, marital and educational status) are presented as absolute (n) and relative (%) frequencies. Normally distributed variables were compared with COVID-19 vaccination acceptance categories using the ANOVA technique, while non-normally distributed variables were compared using the nonparametric Kruskal–Wallis test. The distributions of socio-demographic and work-related characteristics in the different intentions of acceptance of COVID-19 vaccination groups were compared using Pearson's chi-squared test of independence.

Multinomial logistic regression models were used to evaluate the association of previous acceptance of influenza vaccination, receiving the recommended vaccines for health professionals, belonging to a vulnerable group (pregnant, diabetic, immunosuppressed, etc.) where vaccination is recommended, and some work-related factors (i.e., job role, work in a public or private hospital) on intentions of COVID-19 vaccination acceptance adjusted for age and gender. The first model has a dependent variable of intention of COVID-19 vaccination as intended to accept vaccination vs. undecided, while the second model has a dependent variable of intention of COVID-19 vaccination as not intended to accept vaccination vs. undecided. All statistical hypotheses were two sided, with the statistical significance level set at α = 0.05. Statistical analysis was conducted using STATA 14.0 statistical software (Stata Corp, College Station, TX, USA).

## Results

### Participants' Demographics and Work-Related Characteristics

The median age of the 436 participants was 34 (q_1 =_30, q_3 =_42) (Table 1). Most of the study participants were women (71%), residents of Nicosia (46%), married or in cohabitation (75.5%) and had a bachelor's degree (54%). Furthermore, we reported that 93% of the study individuals were nurses, and about 71.5% worked in a public hospital. The most common workplace units were the medical (11%), surgery and orthopedics (11%), intensive care unit (ICU) (10%), and accident and emergency (8.5%) (Table 1). The overall median of the years of experience was 10 (q_1_ = 6, q_3_ = 18). The comparison between the characteristics of our sample and the target population (public or private sector, job role, and city of residence) using information from the Statistical Service of Cyprus (40) was found to be non-significant (p > 0.05).

**Table 1:** Intentions of COVID-19 vaccine acceptance by different participants characteristics.

	**Intention to accept COVID-19 vaccination**				
	**Overall (N = 437)**	**Not intended to accept (*N* = 178)**	**Undecided (*N* = 126)**	**Intended to accept (*N* = 130)**	***p*-Value**
**Gender (*****N*** **=** **435)**^1^					
Male	126 (29.0)	41 (33.1)	29 (23.4)	54 (43.5)	** <0.01**
Female	309 (71.0)	137 (44.5)	96 (31.2)	75 (24.3)	
Age (*N* = 436)^*^	34 (30, 42)	33 (29, 38)	34.5 (30, 43)	36.5 (30, 43)	** <0.01**
**Country (*****N*** **=** **436)**^1^					
Greece	15 (3.5)	6 (40.0)	8 (53.3)	1 (6.7)	0.12
Cyprus	420 (96.5)	171 (41.0)	118 (28.3)	128 (30.7)	
**City (*****N*** **=** **415)**^1^					
Nicosia	191 (46.0)	11 (47.7)	5 (21.7)	7 (30.4)	0.10
Limassol	108 (26.0)	20 (40.0)	16 (32.0)	14 (28.0)	
Larnaca	51 (12.3)	58 (53.7)	25 (23.1)	25 (23.2)	
Paphos	42 (10.2)	63 (33.3)	60 (31.6)	66 (34.9)	
Ammochostos	23 (5.5)	19 (45.2)	13 (31.0)	10 (23.8)	
**Marital status (*****N*** **=** **437)**^1^					
Unmarried	92 (21.1)	40 (44.0)	27 (29.7)	24 (26.3)	0.54
Married/In cohabitation	330 (75.5)	130 (39.5)	95 (28.9)	104 (31.3)	
Divorced/separated/widowed	15 (3.4)	8 (57.1)	4 (28.6)	2 (14.3)	
**Number of children (*****N*** **=** **433)**^1^					
0	152 (35.1)	71 (47.0)	43 (28.5)	37 (24.5)	0.54
1	87 (20.1)	36 (41.9)	24 (27.9)	26 (30.2)	
2	121 (27.9)	45 (37.2)	36 (29.7)	40 (33.1)	
3	55 (12.7)	16 (29.6)	17 (31.5)	21 (38.9)	
4	15 (3.5)	7 (46.7)	3 (20.0)	5 (33.3)	
>5	3 (0.7)	2 (66.7)	0 (0.0)	1 (33.3)	
**Education level (*****N*** **=** **434)**^1^					
Bachelor's degree	235 (53.9)	104 (44.4)	64 (37.4)	66 (28.2)	0.41
Master's degree	186 (42.9)	71 (38.2)	58 (31.2)	57 (30.6)	
PhD	14 (3.2)	3 (23.1)	4 (30.8)	6 (46.1)	
**Job role (*****N*** **=** **435)**^1^					
Midwife	32 (7.4)	14 (43.8)	9 (28.1)	9 (28.1)	0.95
Nurse	403 (92.6)	164 (40.9)	116 (28.9)	121 (30.2)	
**Workplace (*****N*** **=** **437)**^1^					
Surgery and orthopedics	37 (10.8)	11 (29.7)	16 (43.3)	10 (27.0)	0.11
Medical	48 (11.0)	25 (52.1)	13 (27.1)	10 (20.8)	
Intensive care unit (ICU)	42 (9.6)	16 (38.1)	9 (21.4)	17 (40.5)	
Accident and emergency	37 (8.5)	20 (54.1)	5 (13.5)	12 (32.4)	
Maternity	36 (8.2)	16 (44.4)	11 (30.6)	9 (25.0)	
Renal and nephrology	27 (6.2)	12 (44.4)	8 (29.6)	7 (26.0)	
Hematology and oncology	27 (6.2)	11 (40.7)	9 (33.3)	7 (26.0)	
Community unit	20 (4.6)	3 (15.0)	4 (20.0)	13 (65.0)	
Pediatric and pediatric oncology	18 (4.1)	8 (44.4)	6 (33.3)	4 (33.3)	
Isolation ward (COVID-19)	14 (3.2)	3 (21.3)	6 (42.9)	5 (35.8)	
Psychiatric	14 (3.2)	5 (35.8)	4 (28.6)	4 (28.6)	
Other	68 (15.6)	20 (29.4)	25 (36.8)	23 (33.8)	
Not specified	37 (8.9)	18 (48.6)	10 (27.0)	9 (24.4)	
**Public or private hospital (*****N*** **=** **432)**^1^					
Public	309 (71.5)	122 (39.6)	93 (30.2)	93 (30.2)	0.45
Private	123 (28.5)	56 (45.9)	31 (25.4)	35 (28.7)	
**Years of experience (*****N*** **=** 429)^*^	10 (6, 18)	8.5 (5, 15)	11 (7, 20)	13 (7, 20)	** <0.01**

*ICU, intensive care unit*.

* *Median (q_1_, q_3_)*.

1 *Frequency (%). Bold indicates statistically significant at p < 0.05*.

### Demographics and Work-Related Characteristics and Acceptance of COVID-19 Vaccination

Overall, 130 participants (30%) had intentions to accept COVID-19 vaccination, 178 (41%) had no intention to accept, and 126 (29%) were undecided (Table 1). The median age of the 178 participants who had no intention to accept COVID-19 vaccine was 33 (q_1_ = 29, q_3_ = 38), while the median age of the 126 undecided participants and the 130 who intended to accept the vaccine participants was 34.5 (q_1_ = 30, q_3_ = 43) and 36.5 (q_1_ = 30, q_3_ = 43), respectively (*p* < 0.01) (Table 1). Regarding males and females separately, 43.5% of the total number of male participants of the study were intended to accept COVID-19 vaccination, while among the total number of female participants, the corresponding percentage was 24% (*p* < 0.01). In addition, 31% and 23% female and male participants were undecided (*p* < 0.01). In addition, we observed statistically significant differences for years of experience (*p* < 0.01). Specifically, we noticed a larger median number of years of experience among those who intended to accept the vaccine (13) compared with undecided (11) and those who did not intend to accept (8.5) COVID-19 vaccination (*p* < 0.01) (Table 1). We did not find any statistically significant associations among the country of origin, the five geographical areas of Cyprus, the categories of marital and educational status, the number of children, the job role, workplace, and whether they work in the public or private sector.

### Vaccination-Related Intentions, Behaviors, and Attitudes on Intention to Be Vaccinated Against COVID-19

Many participants did not receive a seasonal flu vaccination in the last 5 years (62%). We found statistically significant differences between receiving a seasonal flu vaccination in the last 5 years and intention to accept COVID-19 vaccination. Specifically, among participants who received a seasonal flu vaccination in the last 5 years, most of them (52%) were not intended to accept COVID-19 vaccination (*p* < 0.01). It is important to note that among those who did not receive a seasonal flu vaccination in the last 5 years, no one intended to accept COVID-19 vaccination (Table 2).

**Table 2:** Vaccination-related intentions, behaviors, and attitudes on intention to be vaccinated against COVID-19.

	**Frequency (%)**	**Not intended to accept (*N* = 178)**	**Undecided (*N* = 126)**	**Intended to accept (*N* = 130)**	***p*-Value**
**Received a seasonal flu vaccination in the last 5 years**^**1**^ **(*****N*** **=** **434)**					
Yes	162 (37.3)	141 (52.4)	77 (28.6)	51 (19.0)	** <0.01**
No	269 (62.0)	2 (66.7)	1 (33.3)	0 (0.0)	
I am not sure	3 (0.7)	35 (21.9)	46 (28.7)	79 (49.4)	
**Received the vaccines recommended for health professionals**^**1**^ **(*****N*** **=** **435)**					
Yes	260 (59.8)	89 (66.9)	31 (23.3)	13 (9.8)	** <0.01**
No	133 (30.5)	14 (33.3)	17 (40.5)	11 (26.2)	
I am not sure	42 (9.7)	74 (28.7)	78 (30.2)	106 (41.1)	
**Vaccination at their workplace**^**1**^ **(*****N*** **=** **432)**					
Yes	202 (46.8)	122 (53.0)	64 (27.8)	44 (19.2)	** <0.01**
No	230 (53.2)	53 (26.5)	61 (30.5)	86 (43.0)	
**If they were not vaccinated at their workplace, where they were vaccinated**^**1**^ **(*****N*** **=** **171)**					
Outpatient	58 (33.9)	23 (39.7)	22 (37.9)	13 (22.4)	0.28
Vaccination centers	99 (57.9)	44 (44.4)	28 (28.3)	27 (27.3)	
Personal doctor	9 (5.3)	3 (33.3)	4 (44.4)	2 (22.3)	
Other (e.g., pharmacy, nursing school etc.)	5 (2.9)	1 (20.0)	0 (0.0)	4 (80.0)	
**They belong to a vulnerable group (pregnant, diabetic, immunosuppressed, etc.) to whom vaccination is recommended**^**1**^ **(*****N*** **=** **433)**					
Yes	61 (14.1)	157 (42.4)	106 (28.7)	107 (28.9)	0.40
No	372 (85.9)	21 (34.4)	17 (27.9)	23 (37.7)	
**Awareness of the recommended vaccinations for health professionals**^**1**^ **(*****N*** **=** **435)**					
Yes	286 (65.7)	110 (38.6)	84 (29.5)	91 (31.9)	**0.03**
No	56 (12.9)	34 (60.7)	12 (21.4)	10 (17.9)	
I am not sure	93 (21.4)	33 (35.9)	30 (32.6)	29 (31.5)	
**Attended a conference, seminar, or other training program on vaccination in the last 2 years**^**1**^ **(*****N*** **=** **435)**					
Yes	63 (14.5)	20 (32.3)	18 (29.0)	24 (38.7)	0.43
No	361 (83.0)	153 (42.5)	103 (28.6)	104 (28.9)	
I am not sure	11 (2.5)	5 (45.4)	4 (36.4)	2 (18.2)	
**They recommended and promoted vaccination in their work**^**1**^ **(*****N*** **=** **434)**					
Yes	268 (61.8)	78 (29.2)	77 (28.8)	112 (42.0)	** <0.01**
No	106 (24.4)	64 (60.4)	30 (28.3)	12 (11.3)	
I am not sure	60 (13.8)	35 (59.3)	19 (32.3)	5 (8.5)	
**Nurses and midwives should be vaccinated against COVID-19**^**1**^ **(*****N*** **=** **434)**					
Yes	138 (31.8)	4 (2.9)	19 (13.9)	114 (83.2)	** <0.01**
No	170 (39.2)	137 (80.6)	29 (17.1)	4 (2.3)	
I am not sure	126 (29.0)	36 (28.6)	78 (61.9)	12 (9.5)	
**Intention to accept the COVID-19 vaccine to their children**^**1**^ **(*****N*** **=** **437)**					
Yes	50 (11.7)	0 (0.0)	0 (0.0)	50 (100.0)	** <0.01**
No	185 (42.5)	135 (73.0)	34 (18.4)	16 (8.6)	
I am not sure	94 (21.6)	5 (5.3)	52 (55.3)	37 (39.4)	
I do not have children	105 (24.2)	38 (36.2)	40 (38.1)	27 (25.7)	

1 *Frequency (%). Bold indicates statistically significant at p < 0.05*.

More than half of the participants (60%) received the vaccines recommended for health professionals. We also identified statistically significant differences between receiving the vaccines recommended for health professionals and intention to accept COVID-19 vaccination. The largest number of the participants (41%) who were not sure about receiving the vaccines recommended for health professionals declared their intention to receive COVID-19 vaccination, while the corresponding percentage among those who received the vaccines recommended for health professionals was only 10% (*p* < 0.01). We observed many participants vaccinated at their workplace (47%), and if not, they were vaccinated in vaccination centers (58%) and as outpatients (34%) (Table 2).

Participants who were vaccinated at their workplace stated that the main reasons (total number of reasons reported by the participants) of vaccination was the need for immunization because of their work environment (26%), to protect themselves (25.5%), to protect their family (21%), to protect their patients (15%), because was provided to them for free (6.5%), and because was strongly encouraged in their workplace (6%). Participants selected more than one source of information regarding vaccination (890 responses in total), and the majority identified Internet and social media (35%), colleagues/associates (22%), and educational conferences/seminars as the primary sources of information (17%).

The majority of the participants (66%) were aware of the recommended vaccinations for health professionals, while 61% were not aware and did not intend to accept COVID-19 vaccination (*p* = 0.03). The largest percentage of the participants (42%) who recommend and promote vaccination in their work had intention to accept COVID-19 vaccination. The corresponding percentages for those who did not recommend and promote vaccination in their work or were not sure were 11% and 8.5%, respectively (*p* < 0.01) (Table 2).

When participants questioned whether nurses and midwives should be vaccinated against COVID-19, 39% responded negatively, 32% agreed, and 29% were unsure. Among those who agreed, 83% intended to accept COVID-19 vaccination, while among those who disagreed or were unsure, only 2% and 9.5% intended to accept COVID-19 vaccination, respectively (*p* < 0.01). A large percentage of participants did not accept the COVID-19 vaccination of their children. All the participants, who answered positively to their intention to accept the COVID-19 vaccination of their children, intended to accept a COVID-19 vaccine for themselves (*p* < 0.01) (Table 2).

Among the most common reasons for not accepting the COVID-19 vaccination were concerns regarding the quality and procedures for the approval of the vaccine (expedited development and approval of the vaccine) (38%) and the fear of side effects (31%). Also, 9% of the participants would wait for others to be vaccinated, 8% do not consider themselves to be in a high-risk group, 5% fear a vaccination-related illness, 5% do not consider this disease dangerous, 4% support natural immunization, and 0.3% do not like injection (pain/discomfort).

### Possible Predictors Associated With Intentions to Accept COVID-19 Vaccination

Respondents who received a seasonal flu vaccination in the last 5 years had a higher probability of accepting COVID-19 vaccination. More specifically, participants who received a seasonal flu vaccination in the last 5 years had about two times higher probability of accepting COVID-19 vaccination (Table 3, *adjusted OR* = *2.06, 95% CI: 1.14, 3.74*). We also reported that participants who received a seasonal flu vaccination in the last 5 years had a lower probability of not accepting COVID-19 vaccination (Table 3*, adjusted OR* = *0.54, 95% CI: 0.31, 0.97*). We reported that individuals who received the recommended vaccines for health professionals had 2.46 times higher probability (Table 3, adjusted OR = 2.46, 95% CI: 1.09, 5.53) of accepting COVID-19 vaccination. Furthermore, individuals who work in a private hospital were more likely to accept COVID-19 vaccination than those who work in a public hospital (Table 3, adjusted OR = 2.12, 95% CI: 1.02, 4.37).

**Table 3:** Multinomial logistic regression models for the association between intentions of COVID-19 vaccination acceptance and possible predictors adjusting for age and gender.

**(“Undecided” as reference)**	**Intended to accept vaccination**	**Not intended to accept vaccination**
	**Adjusted OR (95% CI)**	**Adjusted OR (95% CI)**
Age	1.02 (0.98, 1.06)	**0.96 (0.92, 0.99)**
Female	0.39 (0.21, 0.71)	1.22 (0.68, 2.19)
**Received a seasonal flu vaccination in the last 5 years**		
No	Ref	Ref
I do not know	1	0.57 (0.03, 9.90)
Yes	**2.06 (1.14, 3.74)**	**0.54 (0.31, 0.97)**
**Received the vaccines recommended for health professionals**		
No	Ref	Ref
I do not know	1.08 (0.37, 3.16)	**0.28 (0.12, 0.65)**
Yes	**2.46 (1.09, 5.53)**	**0.40 (0.23, 0.69)**
**Vulnerable group (pregnant, diabetic, immunosuppressed, etc.) to whom vaccination is recommended**		
No	Ref	Ref
Yes	1.22 (0.56, 2.64)	1.03 (0.48, 2.20)
**Job role**		
Nurse	Ref	Ref
Midwife	1.61 (0.52, 4.97)	1.10 (0.42, 2.91)
**Public or Private hospital**		
Public	Ref	Ref
Private	**2.12 (1.02, 4.37)**	0.75 (0.39, 1.44)

*Bold indicates statistically significant at p < 0.05*.

## Discussion

Our study, to the best of our knowledge, is the first study investigating the COVID-19 vaccination acceptance among nurses and midwives in Cyprus. We observe that around one-third of the respondents would intend to accept a COVID-19 vaccination; however, around 70% of them were undecided or not intended to accept it and could be qualified as “vaccine hesitant.” Independent factors associated with increased acceptance included gender, age, and years of working experience. Also, we found that having a seasonal flu vaccination in the last 5 years, receiving the vaccines recommended for health professionals, and working at a private hospital associated with a higher probability to accept COVID-19 vaccination.

A low level of intention to accept COVID-19 vaccination and a high ratio of uncertainty on decisions regarding the acceptance of this vaccination was found in our study, despite the current severe situation brought by the COVID-19 pandemic. Hesitancy already exists among nurses regarding routine vaccines, such as seasonal influenza vaccination (42–45). Our results are in agreement with previous studies indicating nurses as a vaccine-hesitant group to receive COVID-19 vaccine (33–36, 46, 47), highlighting the need for suitable actions from public health authorities considering that nurses often interface with patients and frequently are in charge of directly administering vaccines. Of interest, past behavior was one of the main determinants of COVID-19 vaccination, as previous influenza vaccination behavior was found to be a strong predictor of this intention. This finding agrees with a very recent study indicating that nurses who accepted seasonal influenza vaccination in 2019 were more likely to have intentions to accept COVID-19 vaccination when it is available (adjusted OR: 2.03, 95% CI: 1.47–2.81) (33). This suggested that vaccination acceptance is a habit of an individual (48) and underlines the significance of implementing interventions to promote influenza vaccination, which could help improve uptake of COVID-19 vaccination.

The reasons for non-vaccination are complex. Our study identified the main reasons for not receiving the COVID-19 vaccine. The study's participants questioned the quality and procedures for the vaccine approval (38%) and the fear of side effects (31%). The refusal is widely considered the “pandemic public health paradox” due to the low acceptance and uptake of a safe vaccine for such an inflection carrying a high risk (49). However, the effectiveness of vaccination on disease prevention is dependent on the high uptake or coverage rate of the vaccine (50, 51). The high proportion of uncertainty in decisions on the acceptance of COVID-19 vaccination among nurses and midwives could also reflect hesitation around vaccination and a probability of refusal or delay on the vaccination. This could also affect vaccination uptake rate and future vaccination compliance in individuals who engage with vaccine-hesitant nurses on a professional or personal level (17, 36, 52, 53). Social media drive hesitancy, conspiracy theories, and fake news (54, 55). Most of our study participants identified the Internet and social media as the primary sources of vaccination-related information. Those sources could influence their decision to receive or/not the COVID-19 vaccination. Meanwhile, more females, individuals with higher age and a higher number of years of working experience, are likely to accept COVID-19 vaccination compared with those who had no intention to accept vaccination or undecided individuals. The higher likely acceptance of a COVID-19 vaccine in the oldest age group is anticipated as this is the most susceptible group and, therefore, most prone in their self-interest to take this vaccine. Simultaneously, it can be assumed that more experienced nursing staff may witness the positive effects of vaccinations and are therefore less hesitant. Nurses working in the private health sector had higher intention to accept COVID-19 vaccination compared with those in the public sector. Differences in their education and vaccination-related awareness could explain the difference. Another factor could also be that in certain private healthcare units, there is enhanced pressure to follow guidelines, whereas in the public sector, there is no mechanism to enforce vaccination guidelines. However, further investigation is needed to elucidate these assumptions.

Our findings have several implications. The current study results confirm the importance of comprehensive approaches that combine education and recommendation in increasing vaccination rates among nurses and midwives in Cyprus. Our research highlights the necessity of higher compliance among nurses to reach a sufficient COVID-19 vaccine coverage to achieve herd immunity. Efforts should be made to reduce complacency and build vaccination confidence. Therefore, it is essential to educate nurses regarding the benefits of vaccination and the potential health consequences of illness for themselves, their patients, and their family members. Since this population's working schedule includes morning and night shifts, strategies should focus on 24/7 access to vaccination information. Government authorities in Cyprus should ensure that the education is done at a convenient time and place, and therefore, we proposed both small-scale hospital-based seminars and large-scale webinars. Creating a free helpline for general and specific information regarding COVID-19 vaccination for healthcare staff could also be beneficial. Finally, media were identified by different research studies (23, 54, 56) as a huge factor that influences vaccine hesitancy; hence, future campaigns focusing on vaccination safety and efficacy must be made through them.

Some limitations should be considered in interpreting the findings. Our sample was limited to nurses and midwives; therefore, our findings cannot be extrapolated to other healthcare professionals. Moreover, data collection was done using a convenient online survey, limiting our study representativeness. However, an online survey is an alternative approach for data collection in social distancing periods due to the COVID-19 pandemic. Sampling bias may arise as a result of snowball sampling. Another limitation concerns the possibility of bias due to misreporting of self-reported intentions about a hypothetical vaccine. Self-report data could potentially lead to misreporting and information bias, and potential under- or overestimations of reported associations. Also, the cross-sectional design used could not infer a causal relationship. Due to the nature of the questionnaire for assessing individuals' intention to be vaccinated, we choose the answers' option of “yes,” “no,” and “not sure” for participants' convenience; however, the Likert scale could be a better measurement of participants' attitudes. Last, given the hypothetical nature, our results may differ from actual acceptance behaviors. Further studies are needed to compare the behaviors of vaccination during or after the COVID-19 pandemic.

## Conclusion

This is the first study examining the intention of accepting COVID-19 vaccination among nurses and midwives in Cyprus. Our findings reveal that nearly 41% of the participants had no intention to be vaccinated against COVID-19 due to concerns about expedited development and potential side effects. Public health officials and policymakers in Cyprus should implement vaccination promotion strategies to tackle the barriers of COVID-19 vaccine hesitancy. The inadequate levels of vaccine acceptance require immediate efforts to improve vaccination coverage. The nurses' and midwives' workplace enhances the infection risk to them, their family, and the community. Public health authorities in Cyprus should focus on educational programs to address vaccination safety and efficacy concerns, while further studies are required to identify the vaccination coverage after COVID-19 vaccine introduction.

## Data Availability Statement

The raw data supporting the conclusions of this article will be made available by the authors, without undue reservation.

## Ethics Statement

The studies involving human participants were reviewed and approved by Cyprus National Bioethics Committee (CNBC) (EEBK EΠ 2020.01.255). The participants provided their written informed consent to participate in this study.

## Author Contributions

GF conceived and designed the web survey, collected the data, and drafted the original manuscript. MK collected and analyzed the data, and drafted the original manuscript. GT contributed to the design of the study and data collection. KG supervised the study, conceived, designed the web survey, collected the data, and drafted the original manuscript. All authors take responsibility for all aspects of the reliability and freedom from bias of the data presented and the discussed interpretation. All authors read and approved the final manuscript.

## Conflict of Interest

The authors declare that the research was conducted in the absence of any commercial or financial relationships that could be construed as a potential conflict of interest.
