# Supplementary material for: Attitudes and Acceptance of COVID-19 Vaccination Among Nurses and Midwives in Cyprus: A Cross-Sectional Survey
Source: Front Public Health. 2021 Jun 16;9:656138. doi: 10.3389/fpubh.2021.656138 (PMC8244901; doi:10.3389/fpubh.2021.656138)
Supplement: Supplementary file 1 [file Table_1.docx]

| **Item** | **Sign** | **Item-test correlation** | **Item-rest correlation** | **Average interitem covariance** | **Alpha** |
| --- | --- | --- | --- | --- | --- |
| Received a seasonal flu vaccination in the last 5 years | + | 0.6268 | 0.4774 | 0.0419781 | 0.7091 |
| Received the vaccines recommended for health professionals | + | 0.6475 | 0.5011 | 0.0412262 | 0.7053 |
| Vaccination at their workplace | + | 0.6206 | 0.4642 | 0.0420155 | 0.7116 |
| They belong to a vulnerable group (pregnant, diabetic, immunosuppressed, etc.) to whom vaccination is recommended | + | 0.2561 | 0.1141 | 0.053568 | 0.7552 |
| Awareness of the recommended vaccinations for health professionals | + | 0.3766 | 0.1875 | 0.0506144 | 0.7556 |
| Attended a conference, seminar, or other training program on vaccination in the last 2 years | + | 0.2877 | 0.1442 | 0.0531751 | 0.7537 |
| They recommended and promoted vaccination in their work | + | 0.5822 | 0.4208 | 0.0434681 | 0.7185 |
| Nurses and midwives should be vaccinated against COVID-19 | + | 0.7094 | 0.5882 | 0.0395123 | 0.6905 |
| Intention to accept the COVID-19 vaccine | + | 0.6895 | 0.5643 | 0.0402517 | 0.6943 |
| Intention to accept the COVID-19 vaccine to their children | + | 0.6312 | 0.5304 | 0.0442961 | 0.7105 |
| **Test scale** |  |  |  | 0.0450063 | **0.7428** |

**Supplementary Table 1:** Internal reliability using Cronbach’s α-value.
